# Fear Generalization Towards a Stimulus and Context and the Impact of Attention Bias

**DOI:** 10.3390/bs14121230

**Published:** 2024-12-20

**Authors:** Haote Fu, Keying Luo, Zishan Wu, Ruiqi Diao, Xifu Zheng

**Affiliations:** 1 Key Laboratory of Brain, Cognition and Education Sciences, Ministry of Education, South China Normal University, Guangzhou 510663, China; 2022023919@m.scnu.edu.cn (H.F.); 2022023802@m.scnu.edu.cn (K.L.); 2023024013@m.scnu.edu.cn (Z.W.); adiao377@163.com (R.D.); 2 School of Psychology, Center for Studies of Psychological Application, Guangdong Key Laboratory of Mental Health and Cognitive Science, South China Normal University, Guangzhou 510663, China; 3 Center for Studies of Psychological Application, South China Normal University, Guangzhou 510631, China

**Keywords:** conditioned fear, stimulus generalization, contextual generalization, attention control, anxiety

## Abstract

Fear overgeneralization is a prevalent clinical symptom of anxiety disorders. Various research studies have demonstrated that attention plays a crucial role in fear generalization. Moreover, fear is not only generalized to the stimulus, but individuals may also exhibit a certain degree of fear generalization to the context. This research investigates whether fear generalizes to stimuli and context simultaneously and the potential impact of attentional bias. The study involved two conditioned fear factors, a stimulus and context, with visual image materials combining both elements. Participants were instructed to focus on global attention in Study 1, while in Study 2, they were divided into groups based on their attention bias direction towards either stimuli or context during the fear acquisition phase. This study found that participants exhibited generalized conditioned fear to both stimuli and context, regardless of attentional bias. Additionally, participants showed a lower degree of generalization in the area to which they directed their attention during the acquisition phase. The results of this research reveal the differing expressions of fear generalization towards context and stimuli, highlighting the important role of attention in this process.

## 1. Introduction

Fear is a foundational emotion that serves as a vital mechanism for humans and other animals to react to potential threats or dangers. In the face of a threat or uncertainty, fear triggers a stress response that leads to adaptive behaviors [1]. When individuals develop fear towards a particular stimulus, they generally exhibit fear responses not only to that specific stimulus but also to similar ones, a phenomenon referred to as fear generalization [2]. Appropriate fear generalization plays a protective role in an individual’s survival by facilitating swift adaptation to changing environments and avoidance of potential threats. Conversely, excessive fear generalization can negatively impact daily life, leading to significant emotional distress. This exaggerated response constitutes a primary pathological feature of various anxiety disorders [3,4,5,6,7]. Extensive research has been conducted on fear generalization, providing targeted insights into the treatment of anxiety disorders and enhancing the efficacy of interventions for these conditions.

Pavlovian fear conditioning represents a well-established paradigm for the study of fear memory. In the classical conditioned paradigm, a stimulus capable of triggering fear in an individual serves as an unconditioned stimulus (US), while a neutral stimulus serves as the conditioned stimulus (CS). The CS is repeatedly paired with the US to establish a CS–US association that causes a fear response to the CS [8,9]. The discriminative conditioned fear paradigm builds upon the classical conditioning paradigm by introducing two neutral conditioned stimuli. One of the stimuli, as the threatening stimulus (CS+), is paired with the US multiple times, while the other is never matched with the US and is a safe stimulus (CS−) [8].

Fear generalization occurs when stimuli are similar in perception or conceptually related. Individuals’ fear of a CS+ generalizes to perceptually similar stimuli, such as circles of similar sizes, lights of similar colors, or similar human faces [10,11]. This fear generalization is due to individuals’ discrimination bias for perceptually similar features [12]. Furthermore, numerous studies have demonstrated that participants’ fear of a CS+ generalizes to stimuli of related conceptual categories. Stimuli with a high degree of typicality are more likely to elicit a generalized fear response [13]. This is because humans have knowledge and experiences that allow them to make connections between stimuli through cognitive processes like conceptual, categorical, and inductive reasoning [14]. However, the majority of these studies focus on stimulus-based fear generalization. In related studies, additional generalization stimuli (GSs) are introduced that are similar to the CSs. The degree of resemblance between each GS and the CS varies in a gradient, with individuals perceiving the GS that closely resembles the CS+ as more threatening, thus eliciting a stronger fear response [7].

Exposure therapy is a treatment commonly used for anxiety disorders. Its efficacy is also closely related to the patient’s attention [15]. Previous studies have demonstrated that the level of attention exhibited by patients is a significant predictor of the efficacy of exposure therapy [16]. Additionally, training on a dot–probe task during treatment can improve the efficacy of exposure therapy [17]. Research has demonstrated that threatening stimuli are more likely to gain an individual’s attention quicker [18]. Furthermore, some studies have conducted experiments using the paradigm of cue–target. The results showed that individuals exhibit a stronger attentional bias towards threatening stimuli than to neutral stimuli. Furthermore, they indicated that high-anxious individuals have difficulties in attentional disengagement from threats compared to low-anxious individuals [19]. Other studies have also demonstrated that an individual’s level of anxiety also affects their attentional orientation to threatening stimuli [20]. Individuals with high levels of anxiety experience faster attentional orientation to threatening stimuli and also have more difficulty in attentional disengagement from threatening stimuli [21,22]. This suggests that attentional bias is strongly correlated with anxiety.

In addition to a high correlation with anxiety, research has established that attention plays a critical role in the modulation of conditioned fear responses. Becker’s research suggests that deficiencies in attention control may generate anxiety-associated impairments in fear generalization [9]. O’Malley’s experiment revealed that the participants who avoided a stimulus exhibited a significantly higher skin conductance response (SCR) throughout the extinction phase than those in the monitor group and control group. The avoid group also demonstrated a reduced decrease in the SCR towards the CS+ during the extinction retest [23]. These results imply that the avoidance of stimuli is associated with the threat reaction in the process of fear extinction. Additionally, Klein conducted an experiment in which the color of pupils in facial images was manipulated to direct participants’ attention towards the faces during the fear extinction process. The results indicated that participants who maintained focused attention on the threat stimuli exhibited better fear extinction than those in the control group [11].

In addition to stimuli, which exert a crucial influence on fear generalization, context also has an important influence on conditioned fear. Context can be any external or internal information during learning, such as the physical environment or emotional state [24]. A number of researchers have also explored the role of context in fear generalization from this perspective. Background images are mostly employed as context materials in studies [11,25]. When the contexts of fear acquisition and fear extinction differ, conditioned fears, which are successfully extinguished during the extinction phase, may exhibit fear recovery through presentation of the context of the acquisition phase, resulting in increased amygdala activity, an elevated SCR, and an elevated startle response [26].

Conditioned fear also generalizes to the context. A commonly used paradigm is to use a context image that matches the US as the fear context (CTX+) and the context that is not presented simultaneously with the US as the safe context (CTX−). Some background images with similar characteristics to the CTX+ and CTX− were used as the generalization context (GTX), and participants were requested to rate their US expectancy in order to measure the extent of fear generalization towards the context [27]. The results of this study showed that in terms of behavioral responses, individuals exhibited fear towards the GTX, which was similar to results for the CTX+, indicating the presence of fear generalization towards the context. This suggests that individuals not only generalize fear to stimuli but also show fear generalization towards the context. Some researchers have presented contexts in a more realistic manner through virtual reality (VR) technology [27,28,29]. These findings demonstrate that context plays a key role in fear extinction and generalization.

In previous studies, the attentional bias of the participants was guided at the fear extinction phase in order to verify whether attentional bias affects the effect of fear extinction. There is currently no research attempting to verify the impact of manipulating attentional bias on fear generalization. Moreover, a large number of studies have demonstrated that context is also a crucial aspect of information, which has an important influence on an individual’s fear response. In this study, both the stimuli and the context were used as CSs, and they were matched to the US. Building on previous work, participants were instructed to focus on global or local aspects during the fear acquisition phase. The effects of different attentional situations on the conditioned fear stimulus and contextual generalization were explored by comparing physiological responses and behavioral indicators.

We designed two studies in this research to systematically analyze fear generalization towards stimuli and context under different attentional conditions. Two conditioned fear factors, including stimuli and context, were used in the research. Four pictures of conditioned fear materials were presented, as well as two types of generalized pictures. Building upon prior research, we employed instructions [11] and attentional cues [16] to direct participants’ attentional bias, methodologies that have been demonstrated to be effective. In Study 1, participants were instructed to pay attention to global aspects to investigate whether fear generalization occurs together for stimuli and context and whether differences in the degree of their generalization occur under global attention. In Study 2, an attention bias intervention was added. Participants were instructed to pay attention to either the stimulus or the context area to investigate fear generalization under local attention. This research was approved by the Research Ethics Review Board of South China Normal University (Approval Number: SCNU-PSY-2023-229).

The present study hypothesized that under global attention, individuals would generalize fear to both the conditioned stimuli and context, showing stronger fear responses to GSs that are more similar to the CS+. This fear generalization also manifested for the GTXs. With the addition of the attentional bias intervention in Study 2, it was expected that attentional bias would significantly reduce individuals’ fear generalization degree in the biased area. When attention was biased towards a stimulus, individuals would produce a better degree of discrimination of the stimulus and thus show a lower degree of fear generalization. Similarly, when attention was biased towards the context, individuals would exhibit a lower degree of fear generalization to the context.

This research sought to deepen our understanding of the mechanisms involved in fear generalization by examining how attentional bias influences the generalization of fear responses to both stimuli and context. Through this investigation, we aimed to inform future research with more focused insights. Additionally, we aspired for the findings of this study to contribute to the development of more effective therapeutic strategies in clinical settings, thereby establishing an experimental basis for fear overgeneralization and other associated symptoms arising from diverse attentional patterns and conditions.

## 2. Study 1

### 2.1. Methods

#### 2.1.1. Participants

The sample size was determined based on power analyses conducted with G-power [30]. To detect medium effect sizes (Cohen’s f = 0.25) with 90% power for threat level × generalization type ANOVAs, a minimum of 36 participants was required. A total of 46 participants were recruited for the experiment in Study 1. Nine of them were withdrawn early because they failed to pass the threat image discrimination test, implying a failure of fear acquisition. The final valid data were derived from 37 participants, including 12 males, and all of them were university students. Participants were recruited with the requirements that they had no history of any physical or mental disorders, had normal vision or corrected vision, and had no color blindness or color weakness. All participants in the experiment were right-handed and at least 18 years old, and the average age was 19.973 (*SD* = 2.500) years old. Due to the application of electroshock to the participants during the experiment, they were all informed before the experiment that they might receive electric shocks during the experiment and were required to sign an informed consent form. The average shock intensity for the participants in Study 1 was 49.84 volts. The participants received 35 RMB as compensation upon completing the experiment.

The participants were required to finish the State–Trait Anxiety Inventory and the Intolerance of Uncertainty Scale before the formal experiment. The results of the two questionnaires and the characteristics of the participants in this study are summarized in Table 1.

#### 2.1.2. Materials

Visual Images: All visual images in the experiment comprised two distinct components: a stimulus component and a context component. Faces with neutral expressions were used as the stimulus part, one of which served as the CS+ and the other as the CS− (counterbalanced between groups). For the context part, we used two pictures of landscapes recorded by time-lapse photography; one was designated as the CTX+ and the other as the CTX− (counterbalanced between groups). During the experiment, the two stimulus material pictures and the two context material pictures were combined according to a 2 × 2 combination to form four images with a fear condition, including DT (threat stimulus and context), CS+ (threat stimulus with safety context), CTX+ (threat context with safety stimulus), and DS (safety stimulus and context).

**Figure 1:**
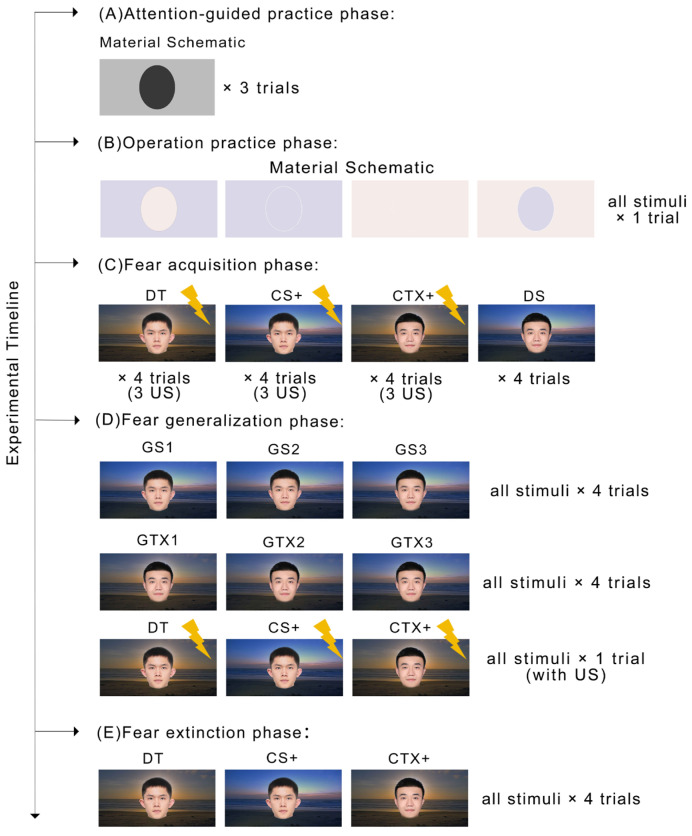
Visual images and experimental timeline. The experiment comprised five phases and utilized a comprehensive set of stimuli, which included four conditioned stimuli (CSs). These were double threat (DS), threat stimulus (CS+), threat context (CTX+), and double safety (DS). Additionally, the experiment involved three generalization stimuli (GSs) and three generalization contexts (GTXs). US refers to the unconditioned stimulus, represented by the yellow lightning bolt in the figure.

Abrosoft FantaMorph 5 software was employed in the synthesis of the generalized images. For the GS, we used the material synthesized from the CS+ and CS− as the stimulus component and CTX− as the context component, while GTX used the material synthesized from the CTX+ and CTX− as the context component and the CS− as the stimulus component. To ensure that the participants had the same discrimination ability for GSs and GTXs at the same level of generalization, a discrimination ability assessment was conducted prior to the experiment. A total of 31 participants were included in the assessment, resulting in the selection of stimulus generalization pictures with a 24% difference gradient (mean discrimination: 50.27%) and contextual generalization pictures with an 18% difference gradient (mean discrimination: 53.70%). There were no significant differences between participants in discrimination rates for the stimulus generalization pictures and contextual generalization pictures that we selected, *t* (30) = 0.260, *p* = 0.797, *d* = 0.095, 95% CI [−0.113, 0.146]. Thus, GS1–3 consisted of the CTX− and stimulus generalization pictures with a 24% difference gradient, while GTX1–3 consisted of the CS− and context generalization pictures with an 18% difference gradient. Visual images are shown in Figure 1.

Electroshock: In this research, electroshock was employed as the US and administered via a Digitimer DS2A isolated voltage stimulator (Hertfordshire, UK). The electric shock was applied to the right wrist of each participant, and the intensity of the shock was rated verbally by the participant before the experiment on a scale ranging from 1 (no sensation at all) to 9 (completely intolerable). The final intensity used was that rated by the participant as 8 (extremely uncomfortable but still tolerable). If a trial required a US to appear, an electroshock was triggered 5.5 s after the images were presented, with each lasting 200 ms.

#### 2.1.3. Procedure

The experimental procedure was edited using E-prime 3.0. The experiment commenced with an attention-guided practice phase, followed by an operation practice phase, a fear acquisition phase, a fear generalization phase, and a fear extinction phase. Subsequent to the completion of two questionnaires, the participants proceeded to the formal experiment.

Attention-guided practice phase: To ensure effective manipulation of participants’ attentional bias, the first phase was intended to help them develop the habit of focusing on the global aspects of the images. This phase included three trials. In each trial, the attention cue picture was presented first for a duration of 2 s, followed by a schematic diagram for 3 s, and finally a black screen for 3 s. The attention cue picture consisted of only the white square portion in Study 1, and the schematic distinguished stimulus and context areas through dark and light grey. The participants were required to focus on the white square portion of the attention cue image and to maintain this attentional habit while the schematic diagram was presented.

Operation practice phase: The purpose of this phase was to enable participants to be familiar with the key operation. In order to prevent early presentation of the images, particularly the context, before fear acquisition from affecting the subsequent process, the schematic diagram was still used in this phase, which indicated the location of the stimulus and the context areas by purple and pink (or yellow and green) of the same lightness, saturation, and color hue. A total of one block included four trials.

**Figure 2:**
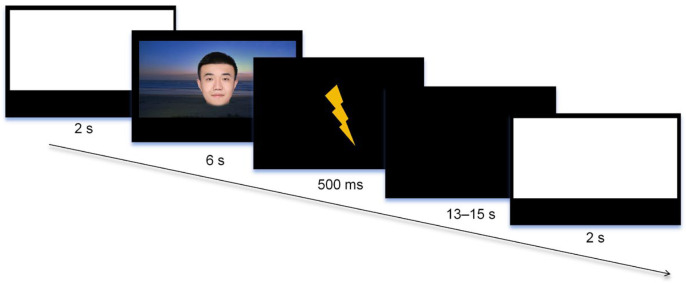
Schematic representation of image presentation during the experiment.

In each trial, an attention cue picture was presented for 2 s, followed by a schematic diagram for 6 s, Thereafter, there was an inter-trial interval (ITI) of a random duration between 13 s and 15 s. The participants needed to rate US expectancy while the schematic diagram was displayed. When the schematic diagram was presented, the text “What do you think is the likelihood of a subsequent electric shock?”, and a rating bar with “1” for “not at all” and “9” for “definitely” appeared below. The participants were asked to press a key from 1 to 9 on the numeric keypad within 5 s, with larger numbers indicating that the participant thought that a shock was more likely to follow after that stimulus. The same timeline was employed in each trial during the subsequent phase, as shown in Figure 2.

Fear acquisition phase: The purpose of this stage was to establish conditioned fear associations. It was divided into two blocks, and in each block, the DT, CS+, CTX+, and DS were presented two times, for a total of 16 trials. The DT, CS+, and CTX+ were presented with a 75% probability of the US. Visual images were presented in a pseudorandom manner in the formal experiment.

To ensure the validity of the data, the participants were required to take a threat image discrimination test after the acquisition phase to confirm their successful acquisition of fear. The participants were presented with the DT, CS+, CTX+, and DS and were guided to press a key by the instruction “Which of the following pictures is safe without triggering an electroshock at all?”. Once the participants chose correctly, they entered the fear generalization phase. If a participant chose incorrectly, the experiment ended.

Fear generalization phase: In this phase, there were two blocks, and in each block, each of six generalized images was presented two times, for a total of 24 trials. To avoid participants developing fear extinction in the generalization phase, the DT, CS+, and CTX+ were presented one time each with the US in this phase.

Fear extinction phase: The aim of this phase was to reduce fear of the CS. It was divided into two blocks, with the DT, CS+, and CTX+ presented two times each in each block for total 12 trials. No US was presented in this phase.

### 2.2. Data Recording and Processing

#### 2.2.1. US Expectancy

Behavioral responses were collected during every trial with a keyboard. The participants expressed their subjective expected value of the US using the numbers 1 to 9. Missing values were replaced with the mean for that phase.

#### 2.2.2. Skin Conductance Response (SCR)

Experiments were conducted using a BIOPAC MP150 for SCR data acquisition at a sampling rate of 1000 Hz. During the experiment, Ag/AgCl electrodes were attached to the terminal phalanges of the index and ring fingers of the participant’s left hand.

AcqKnowledge 4.2 software was used for SCR data acquisition and processing. The SCR for each trial was obtained by subtracting the averaged skin conductance level during 2 s prior to image onset from the highest value recorded within 5.5 s after image onset. SCRs below 0.02 μS were replaced with 0. For each individual, these amplitudes were then range-corrected using the largest response elicited by the US as the maximum range. All SCRs were then transformed by the square root to reduce skewness [31].

#### 2.2.3. Generalization Index (GI)

In the present research, the generalization index (GI) of each generalization image was included in the analysis of the data from the fear generalization phase to account for potential differences in fear acquisition between participants in different attentional conditions. The indices were denoted as GI-GSi (i = 1, 2, 3) and GI-GTXi (i = 1, 2, 3), where GI-GSi represents the GI for each GS, and GI-GTXi represents the GI for each GTX.

The formulas were GI-GSi = GSi/CS + (i = 1, 2, 3) and GI-GTXi = GTXi/CTX+ (i = 1, 2, 3). 

In endocrinological research, the degree of response to a continuous measure is often obtained from the value of the area under the curve for each set of numbers [32]. A similar calculation has been used in studies of fear generalization to indicate the degree of participants’ generalization through the GI [33,34], which is calculated as GI = [GS1 + GS2 + GS3 + GS4]/CS+. In this study, we aimed to obtain insights into how individuals generalize fear to a stimulus and context under different attention conditions, which can be intuitively measured through the generalization gradient. We calculated the sum of the GIs for each generalization category and obtained the degree of generalization for the stimulus area and context area separately and thus for the differences between the different types of generalization. The indices were denoted as GI-GS and GI-GTX, respectively.

The formulas were GI-GS = [GS1 + GS2 + GS3]/CS+ and GI-GTX = [GTX1 + GTX2 + GTX]/CTX+.

### 2.3. Statistical Analysis

In the fear acquisition phase, an image type (DT, CS+, CTX+, DS) × block (BLOCK1, BLOCK2) two-factor repeated-measures ANOVA was performed. We determined whether the participants developed fear generalization by comparing the DS results of BLOCK2 in the acquisition phase with the GS1–3 results and GTX1–3 results through two one-way repeated-measures ANOVAs, respectively. During the fear generalization phase, the GI for each generalization image was included in a two-factor repeated-measures ANOVA to analyze the effects of threat level (high, middle, low) and generalization type (GS, GTX). The GI for generalization degree was included in a paired-samples *t*-test as well to compare the degree of generalization (GI-GS, GI-GTX). Additionally, SCR results from BLOCK1 of the generalization phase were included in the data analysis. A two-factor repeated-measures ANOVA for image type (DT, CS+, CTX+) and block (BLOCK1, BLOCK2) was conducted during the fear extinction phase.

Data analysis for this study was performed using SPSS 25 software.

### 2.4. Result

#### 2.4.1. Fear Acquisition

**Figure 3:**
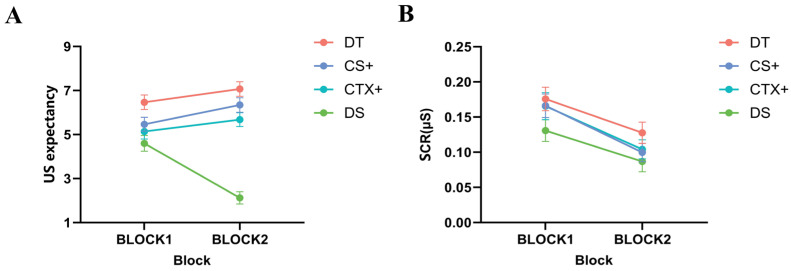
Differences in US expectancy and skin conductance responses (SCRs) during fear acquisition in Study 1. During the fear acquisition phase, both (**A**) US expectancy and (**B**) SCRs were significantly higher for the DT, CS+, and CTX+ than for the DS. Standard error bars are presented.

US expectancy: In the results of the two-factor repeated-measures ANOVA for image type (DT, CS+, CTX+, DS) × block (BLOCK1, BLOCK2), the main effect of image type was significant, *F* (3, 108) = 38.304, *p* < 0.001, $\eta_{p}^{2}\;$ = 0.516. Post-hoc tests showed that participants rated the DT significantly higher than the CS+ (*MD* = 0.864, *SE* = 0.368, *p* = 0.025, 95% CI [0.117, 1.611]) and significantly higher than the CTX+ (*MD* = 1.360, *SE* = 0.302, *p* < 0.001, 95% CI [0.749, 1.972]). The interaction between image type and block was significant, *F* (3, 108) = 16.669, *p* < 0.001, ${\;\eta }_{p}^{2}$ = 0.316. Post-hoc test showed that the US expectancy of the CS+ was significantly higher in BLOCK2 than in BLOCK1 (*MD* = 0.878, *SE* = 0.423, *p* = 0.045, 95% CI [0.021, 1.736]), and the US expectancy of the DS was significantly lower in BLOCK2 than in BLOCK1 (*MD* = 2.477, *SE* = 0.313, *p* < 0.001, 95% CI [1.843, 3.111]). The results indicated that the participants successfully acquired fear, were significantly more afraid of the DT, CS+, and CTX+ than the DS, and were more intensely afraid of the DT with double-threat factors (see Figure 3A).

SCR: In the results of the two-way repeated-measures ANOVA for image type (DT, CS+, CTX+, DS) × block (BLOCK1, BLOCK2), there was a significant main effect of image type, *F* (3, 108) = 3.550, *p* = 0.017, ${\;\eta }_{p}^{2}$ = 0.090. A significant main effect of block, *F* (1, 36) = 26.953, *p* < 0.001, ${\;\eta }_{p}^{2}$ = 0.428, showed that the participants had significantly higher SCRs in BLOCK1 than in BLOCK2. Because SCRs were less sensitive and more likely to adapt to novel stimuli, there was no interaction between stage and stimulus type compared to US expectancy (see Figure 3B).

#### 2.4.2. Fear Generalization

US expectancy: A significant main effect of image type was found in the results of the one-way repeated-measures ANOVA. Significant differences were found between DS and GS1–3, as well as between DS and GTX1–3, with *Fs* ≥ 41.484, *ps* ≤ 0.001, suggesting that fear generalization occurred in the participants after fear acquisition.

**Figure 4:**
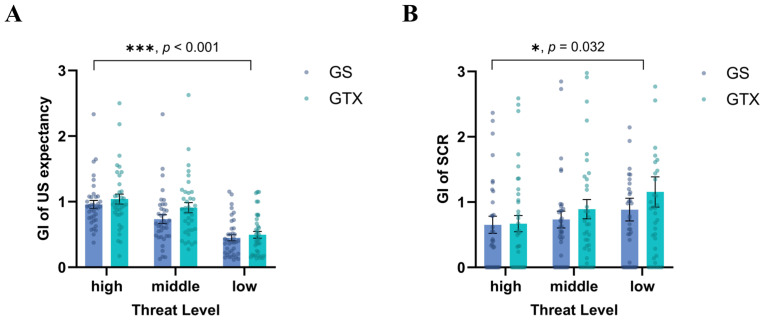
Differences in US expectancy and SCRs during fear generalization in Study 1. (**A**) US expectancy was significantly higher for the generalization image with a high-threat level and lower for the low-threat-level image. (**B**) SCRs were significantly higher for generalization images with a low-threat level and lower for the high-threat-level image. Standard error bars are presented.

In the fear generalization phase, a two-way repeated-measures ANOVA for threat level (high, middle, low) × generalization type (GS, GTX) showed a significant main effect of threat level, *F* (2, 72) = 72.414, *p* < 0.001, $\eta_{p}^{2}$ = 0.668. In the post-hoc test results, the GIs of the high-threat level were significantly higher than those of the middle-threat level (*MD* = 0.178, *SE* = 0.028, *p* < 0.001, 95% CI [0.121, 0.235]). The GIs of the middle-threat level were significantly higher than those of the low-threat level (*MD* = 0.526, *SE* = 0.053, *p* < 0.001, 95% CI [0.420, 0.633]). The GI of the generalized images increased as the threat level of the images increased, suggesting that the participants developed graded fear generalization. The paired-samples *t*-test results for the degree of generalization did not reveal any statistically significant differences between stimuli and context, *t* (36) = 1.404, *p* = 0.169, *d* = 0.231, 95% CI [0.134, −0.739] (see Figure 4A).

SCR: In the results of the one-way repeated-measures ANOVA, no significant difference was found between the DS and generalization images, with *Fs* ≤ 2.392, *ps* ≥ 0.073. A two-way repeated-measures ANOVA for threat level (high, middle, low) × generalization type (GS, GTX) revealed a significant main effect of threat level, *F* (2, 72) = 3.615, *p* = 0.032, $\eta_{p}^{2}$ = 0.091. Post-hoc tests revealed that the participants had a significantly lower GI for the high-threat level than for the low-threat level (*MD* = −0.359, *SE* = 0.154, *p* = 0.025, 95% CI [−0.671, −0.047]). Similar to the results for US expectancy, the results of the paired-samples *t*-tests for the degree of generalization showed no significant differences between stimuli and context, *t* (36) = 0.933, *p* = 0.357, *d* = 0.153, 95% CI [1.416, −0.524] (see Figure 4B).

#### 2.4.3. Fear Extinction

**Figure 5:**
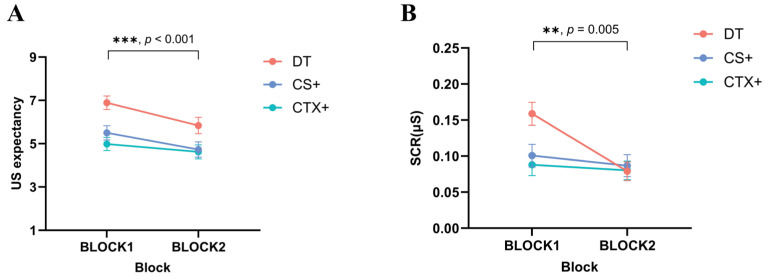
Differences in US expectancy and SCRs during fear extinction in Study 1. Both (**A**) US expectancy and (**B**) SCRs significantly decreased during fear extinction, and the result for the DT was significantly higher than those for the other image types. Standard error bars are presented.

US expectancy: The results of the two-factor repeated-measures ANOVA for image type (DT, CS+, CTX+) × block (BLOCK1, BLOCK2) showed a significant main effect of image type, *F* (2, 72) = 14.494, *p* < 0.001, $\eta_{p}^{2}$ = 0.287. The post-hoc test showed that the participants rated the DT significantly higher than the CS+ and CTX+ (*MD* = 1.250, *SE* = 0.335, *p* = 0.001, 95% CI [0.570, 1.930]; *MD* = 1.561, *SE* = 0.295, *p* < 0.001, 95% CI [0.962, 2.160]). The main effect of the block was significant, *F* (1, 36) = 27.495, *p* < 0.001, $\eta_{p}^{2}$ = 0.433. US expectancy was significantly higher in BLOCK1 than in BLOCK2, suggesting that the participants were successful in the extinction of fear at this phase (see Figure 5A).

SCR: The results of the two-factor repeated-measures ANOVA for image type (DT, CS+, CTX+) × block (BLOCK1, BLOCK2) on SCRs were similar to the results for US expectancy. The main effect of image type was significant, *F* (2, 72) = 4.002, *p* = 0.020, $\eta_{p}^{2}$ = 0.100. Post-hoc tests showed that the participants’ SCRs to the DT were marginally significantly higher than the SCRs to the CS+ (*MD* = 0.025, *SE* = 0.013, *p* = 0.052, 95% CI [0.000, 0.051]) and significantly higher than the SCRs to the CTX+ (*MD* = 0. 035, *SE* = 0.014, *p* = 0.016, 95% CI [0.007, 0.063]). The main effect of the block was significant, *F* (1, 36) = 9.028, *p* = 0.005, $\eta_{p}^{2}$ = 0.201, and the participants had significantly higher SCRs in BLOCK1 than in BLOCK2. The interaction effect of image type and phase was significant, *F* (2, 72) = 6.985, *p* = 0.002, $\eta_{p}^{2}$ = 0.162. The participants were significantly more fearful of the DT than of the CS+ and CTX+ in BLOCK1 (*MD* = 0. 058, *SE* = 0.016, *p* = 0.001, 95% CI [0.025, 0.091]; *MD* = 0.071, *SE* = 0.031, *p* = 0.002, 95% CI [0.028, 0.113]), but no significant differences between the three image types were found in BLOCK2 (see Figure 5B).

### 2.5. Discussion

During the fear acquisition phase, both in terms of US expectancy and SCRs, the fear responses exhibited by the participants to the DT, CS+, and CTX+ were significantly higher than those to the DS. The interaction between image type and block was also significant. This evidence demonstrated successful acquisition of fear. Furthermore, the results indicated that the US expectancy for the DT was significantly higher than that for the other images, suggesting a stronger fear response to double-threat factors. The same difference was also evident in the process of fear extinction, where the fear responses of the participants to the DT were significantly higher than those to the other images. This may be attributed to the fact that when the DT was presented, the participants were under multiple threats, and their emotions were more aroused, resulting in stronger fear responses. It is also possible that double-threat stimuli can elicit a more significant cognitive load from individuals, and previous research has shown that individuals pay greater attention to threat cues when they are under a high cognitive load [35], which results in stronger fear responses.

In the process of fear generalization, the results of the present study were consistent with the expectation that individuals exhibited fear generalization towards both the stimuli and the context. Previous studies have indicated that the level of US expectancy increases as the level of threat increases, resulting in fear generalization [8,36,37,38]. The results of previous studies on contextual generalization are also comparable to the results of fear generalization towards stimuli, with participants showing stronger fear responses to contexts similar to the CTX+, thereby demonstrating fear generalization towards the context [25,27,28,39]. In the present study, two conditioned fear factors, the stimulus and the context, were set up simultaneously in order to explore the generalization of fear for two factors at the same time in a within-group format. The results demonstrated that both the stimulus and the context factors contributed to fear generalization, which was likely due to the similarity of the threat factors.

However, the present study demonstrated that the SCRs of the participants for the generalized images appeared to increase with a decrease in the threat level. The SCRs produced for the high-threat-level generalization images were significantly lower than those for the middle-threat level and low-threat level, which appeared to be inconsistent with US expectancy. The separation of physiological and behavioral data has also been observed in other studies. Dual-process Theories (DPT) may explain this phenomenon. The theory posits the reasons for the existence of two distinct learning systems to facilitate the acquisition of CS–US conditioned reflexes. One is an explicit learning system based on a system of rules. The other is an implicit learning system that does not require conscious involvement. The explicit learning process is associated with US expectancy, while the SCR reflects the result of implicit learning [40,41]. The separation of subjective and objective indicators identified in the present study suggests that individuals exhibit a fear of high-threat-level generalization images at the conscious episodic level. At the unconscious implicit level, participants with global attention may focus more clearly on the pairing of a stimulus and context. Participants will be more unfamiliar with middle-threat- and low-threat-level generalization images due to changes in the pairing, leading to higher SCRs.

Otherwise, no significant differences in generalization were observed between the stimulus and the context. This may be attributed to the fact that individuals are able to discriminate both a stimulus and context to an equal degree when they are engaged in global attention. Struyf explored differences between perceptual discrimination of stimuli and the degree of fear generalization [4]. His finding demonstrated that participants with varying perceptual discrimination abilities exhibited different degrees of generalization. Consequently, the findings of the present study may be associated with the material discrimination ratings, where the selected stimuli and contexts elicited comparable discriminative rates in the selection of the generalization images.

## 3. Study 2

The findings of Study 1 indicate that individuals under global attention exhibit similar fear generalization degrees to both stimuli and context. We further conducted Study 2 to verify that the degree of fear generalization towards stimuli and context may be influenced by attentional bias. Consequently, the design was refined to include guidance on attentional bias during the acquisition phase based on the assumption that individuals would be better able to discriminate the area to which their attention was biased, resulting in a milder degree of fear generalization.

### 3.1. Methods

The materials and apparatus utilized in Study 2 were identical to those employed in Study 1.

#### 3.1.1. Participants

According to the results of the G-power analyses, a minimum of 46 participants was necessary to achieve 90% power for detecting medium effect sizes (Cohen’s f = 0.25) in the ANOVAs analyzing generalization degree by group. A total of 81 participants were recruited for the experiment in Study 2. Eight of them were withdrawn early because they failed to pass the threat stimulus discrimination test, which implies failure of fear acquisition. The final valid data set consisted of 73 participants randomly divided into two groups; the stimulus bias group was guided to pay attention to the stimulus area, and the context bias group was guided to pay attention to the context area. The stimulus bias group included 31 participants, 12 of whom were male, and their average age was 20.56 (*SD* = 2.24) years old. The context bias group included 32 participants, 10 of whom were male, and their average age was 20.66 (*SD* = 2.09) years old. The average shock intensity for the stimulus bias group was 53.48 volts, while that for the context bias group was 56.97 volts. As in Study 1, the participants were requested to complete the State-Trait Anxiety Inventory and the Intolerance of Uncertainty Scale before the experiment. The demographic information and questionnaire results are presented in Table 2.

#### 3.1.2. Procedure

The methodology used in Study 2 was almost the same as that used in Study 1, including the following phases: the attention-guided practice phase, operation practice phase, fear acquisition phase, fear generalization phase, and fear extinction phase. In Study 2, the attention cue pictures were different, with the aim to manipulate attention bias. The stimulus group was shown the picture with a white square portion in the stimulus area, and the context group saw the picture with that white square portion in the context area. Participants were instructed to rate valence and arousal towards four conditioned images after the fear acquisition phase and after the extinction phase in this study. Moreover, the extinction phase in Study 2 was divided into three blocks, with each block comprising two presentations of the DT, CS+ and CTX+ for a total of 18 trials.

### 3.2. Data Recording and Processing

In Study 2, US expectancy and SCRs were also recorded. We also calculated the GIs for the data from the generalization phase. Additionally, the valence and arousal ratings of the conditioned images were included.

#### Valence and Arousal Ratings

In reference to O’Malley and Waters’ study [23], we established valence and arousal ratings within the experiment. After the fear acquisition phase and fear extinction phase, the participants were asked to rate their valence and arousal towards the images that appeared. This rating was obtained through a self-assessment mannequin (SAM), which allowed the participants to subjectively evaluate the fear-conditioned images [16]. The assessment was conducted on a 9-point scale, with the participants expressing their subjective feelings using a numeric keypad. Valence towards images was rated on a scale from 1 (very pleasant) to 9 (very unpleasant). Arousal towards images was rated on a scale from 1 (very calm) to 9 (very excited).

### 3.3. Statistical Analysis

A three-factor repeated-measures ANOVA was conducted to assess the acquisition of fear. The factors included image type (DT, CS+, CTX+, DS), block (BLOCK1, BLOCK2), and group (stimulus bias, contextual bias). An image type (DS, GS1–3) × group (stimulus bias, context bias) two-way repeated-measures ANOVAs and an image type (DS, GTX1–3) × group (stimulus bias, context bias) two-way repeated-measures ANOVAs were conducted in order to determine if participants exhibited fear generalization. As in Study 1, GIs were used for the analysis of fear generalization results. We conducted a three-way repeated-measures ANOVA of threat level (high, middle, low) × generalization type (GS, GTX) × group (stimulus bias, context bias) and a repeated-measures ANOVA of generalization degree (GI-GS, GI-GTX) × group (stimulus bias, context bias). For the analysis of SCR results in the generalization phase, only the BLOCK1 results were included. In the fear extinction phase, a three-way repeated-measures ANOVA of image type (DT, CS+, CTX+) × group (stimulus bias, context bias) × block (BLOCK1, BLOCK2, BLOCK3) was conducted. A two-way repeated-measures ANOVA was conducted with image type (DT, CS+, CTX+, DS) × rating time (post-acquisition, post-extinction) for the ratings of valence and arousal.

### 3.4. Results

#### 3.4.1. Fear Acquisition

**Figure 6:**
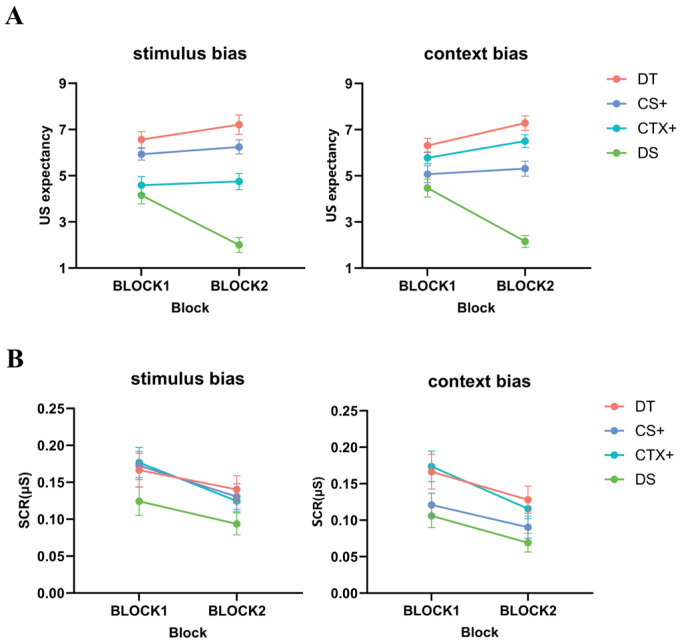
Differences in US expectancy and SCRs during fear acquisition in Study 2. During fear acquisition, all participants showed significantly higher (**A**) US expectancy and (**B**) SCRs for the DT, CS+, and CTX+ than for the DS. Standard error bars are presented.

US expectancy: A three-way repeated-measures ANOVA for image type (DT, CS+, CTX+, DS) × group (stimulus bias, context bias) × block (BLOCK1, BLOCK2) revealed a significant main effect of image type, *F* (3, 183) = 52.215, *p* < 0.001, $\eta_{p}^{2}$ = 0.461, and a significant interaction effect between image type and group, *F* (3, 183) = 8.263, *p* < 0.001, $\eta_{p}^{2}$ = 0.119. The post-hoc test results showed that the stimulus bias group had significantly higher US expectancy for the CS+ than the context bias group (*MD* = 0.899, *SE* = 0.368, *p* = 0.017, 95% CI [0.163, 1.634]). Furthermore, the context bias group rated the CTX+ significantly higher than the stimulus bias group rated the CTX+ (*MD* = 1.469, *SE* = 0.348, *p* < 0.001, 95% CI [0.773, 2.165]). There was a significant interaction effect between image type and block, *F* (3, 183) = 52.214, *p* < 0.001, $\eta_{p}^{2}$ = 0.456. The post-hoc test results indicated that participants’ US expectancy for the DT in BLOCK2 was significantly higher than that in BLOCK1 (*MD* = 2.936, *SE* = 0.398, *p* < 0.001, 95% CI [−3.731, −2.140]), while their US expectancy for the DS in BLOCK2 was significantly lower than that in BLOCK1 (*MD* = 2.232, *SE* = 0.321, *p* < 0.001, 95% CI [1.590, 2.873]). These results suggest that the participants in both groups successfully acquired fear. Additionally, under different attentional bias interventions, the participants provided different US expectancy ratings for the CS+ and CTX+, demonstrating the effectiveness of the attentional bias intervention method used in this experiment (see Figure 6A).

SCR: A three-way repeated-measures ANOVA for image type (DT, CS+, CTX+, DS) × group (stimulus bias, context bias) × block (BLOCK1, BLOCK2) revealed a significant main effect of image type, *F* (3, 183) = 7.803, *p* < 0.001, $\eta_{p}^{2}$ = 0.113. Post-hoc test results showed that SCRs for the DS were lower than those for the DT, CS+, and CTX+ (*MD* = −0.052, *SE* = 0. 012, *p* < 0.001, 95% CI [−0.076, −0.028]; *MD* = −0.030, *SE* = 0.010, *p* = 0.004, 95% CI [−0.050, −0.010]; *MD* = 0.049, *SE* = 0.011, *p* < 0.001, 95% CI [−0.071, −0.028]). The main effect of block was significant, *F* (1, 61) = 32.158, *p* < 0.001, $\eta_{p}^{2}$ = 0.345, with the participants exhibiting significantly greater SCRs in BLOCK1 than in BLOCK2 (*MD* = 0.039, *SE* = 0.007, *p* < 0.001, 95% CI [−0.025, −0.053]) (see Figure 6B).

#### 3.4.2. Fear Generalization

**Figure 7:**
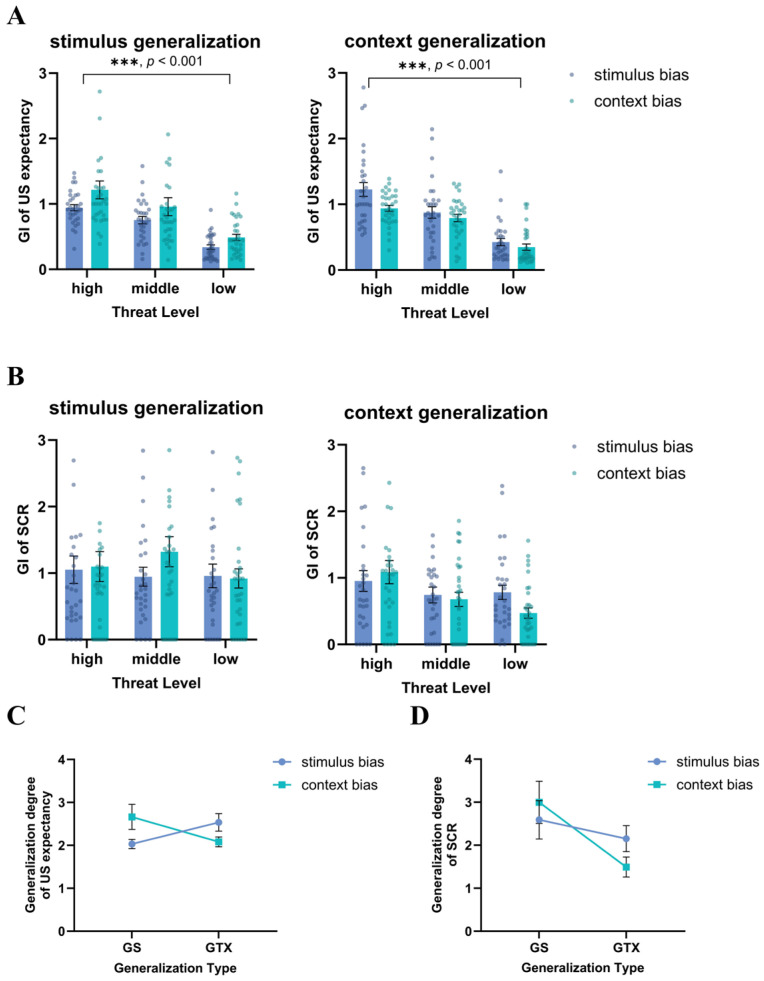
Differences in the generalization index (GI) of US expectancy and the GI of the SCR during fear generalization in Study 2. (**A**) US expectancy was significantly higher for the generalization image with a high-threat level and lower for the low-threat-level image. (**B**) SCR were not significantly different across threat levels. All participants exhibited elevated SCRs for the GS. The stimulus bias group showed a lower degree of stimulus generalization, while context bias group got a lower degree of contextual generalization in both GI of (**C**) US expectancy and (**D**) SCR. Standard error bars are presented.

US expectancy: The results of two-way repeated-measures ANOVAs demonstrated that fear generalization occurred in the participants after fear acquisition. All main effects of image type were significant, *Fs* ≥ 97.105, *ps* ≤ 0.001. In the analysis with the GI of each generalization image, a repeated-measures ANOVA for threat level (high, middle, low) × generalization type (GS, GTX) × group (stimulus bias, context bias) revealed a significant main effect of threat level, *F* (2, 122) = 127.357, *p* < 0.001, $\eta_{p}^{2}$ = 0.676, with the participants having a significantly higher GI for the high-threat level than the middle-threat level (*MD* = 0.236, *SE* = 0.031, *p* < 0.001, 95% CI [0.174, 0.298]). Furthermore, the GI for the middle-threat level was also significantly higher than that for the low-threat level (*MD* = 0.444, *SE* = 0.045, *p* < 0.001, 95% CI [0.353, 0.535]). The interaction between generalization type and group was significant, *F* (1, 61) = 9.354, *p* = 0.003, $\eta_{p}^{2}$ = 0.133. Similarly, the interaction between threat level, generalization type, and group was significant, *F* (2, 122) = 3.289, *p* = 0.046, $\eta_{p}^{2}$ = 0.051. The results of the post-hoc test indicated that the participants in the context bias group exhibited a significantly higher GI for GS3 than those in the stimulus bias group (*MD* = 0.149, *SE* = 0.059, *p* = 0.013, 95% CI [0.032, 0.267]). Conversely, the GI of GTX1 in the stimulus bias group was significantly higher than that in the context bias group (*MD* = 0.287, *SE* = 0.113, *p* = 0.013, 95% CI [0.062, 0.513]). In a repeated-measures ANOVA for generalization degree (GI-GS, GI-GTX) × group (stimulus bias, context bias), the interaction between generalization type and group was significant, *F* (1, 61) = 9.471, *p* = 0.003, $\eta_{p}^{2}$ = 0.134. The results of the post-hoc test indicated that the stimulus bias group generalized at a significantly lower degree for the GS than the context bias group (*MD* = −0.631, *SE* = 0.315, *p* = 0.049, 95% CI [−1.261, −0.001]), and the context bias group generalized the GTX to a marginally significantly lower degree than the stimulus bias group (*MD* = −0.453, *SE* = 0.232, *p* = 0.055, 95% CI [−0.916, −0.010]). The results can be combined to reveal that individuals exhibited varying degrees of fear generalization towards the stimulus and the context under various conditions of attentional bias. For the participants who paid attention to the stimulus, the degree of generalization produced towards the GS was significantly lower than that towards the GTX, suggesting better discrimination of the stimulus. Conversely, the participants who paid attention to the context exhibited a lower degree of generalization towards the GTX, and they were better discriminators of the context (see Figure 7A).

SCR: In the results of two-way repeated-measures ANOVAs, the main effect of image type was significant, *Fs* ≥ 3.160, *ps* ≤ 0.026, and fear generalization occurred in the participants after acquisition. The results of the three-way repeated-measures ANOVA for threat level (high, middle, low) × generalization type (GS, GTX) × group (stimulus bias, context bias) demonstrated a significant interaction between generalization type and threat level, *F* (2, 122) = 4.471, *p* = 0.012, $\eta_{p}^{2}$ = 0.068. The results of the post-hoc tests for GS1 vs. GTX1 and GS2 vs. GTX2 did not reveal any significant differences. However, the participants exhibited a significantly higher GI for GS3 than for GTX3 (*MD* = 0.798, *SE* = 0.325, *p* = 0.017, 95% CI [0.149, 1.447]). In a repeated-measures ANOVA for generalization degree (GI-GS, GI-GTX) × group (stimulus bias, context bias), a significant main effect of generalization degree was observed, *F* (1, 61) = 7.085, *p* = 0.010, $\eta_{p}^{2}$ = 0.104. Both the stimulus bias group and the context bias group demonstrated a greater degree of fear generalization towards the GS. The above results indicate that the degree of generalization towards the GS by the participants was significantly higher than that towards the GTX (see Figure 7B).

#### 3.4.3. Fear Extinction

**Figure 8:**
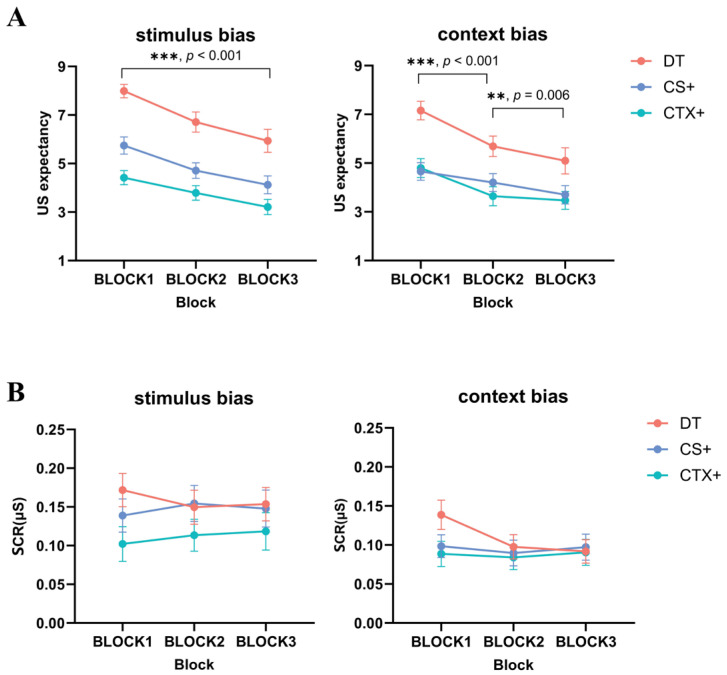
Differences in US expectancy and SCRs during fear extinction in Study 2. During fear acquisition, the (**A**) US expectancy and (**B**) SCR results of all participants were significantly decreased during fear extinction, and the results for the DT were significantly higher than those for the other image types. Standard error bars are presented.

US expectancy: The results of a three-way repeated-measures ANOVA for image type (DT, CS+, CTX+) × group (stimulus bias, context bias) × block (BLOCK1, BLOCK2, BLOCK3) demonstrated a significant main effect of image type, *F* (2, 122) = 54.415, *p* < 0.001, $\eta_{p}^{2}$= 0.476, and the main effect of the block was also significant, *F* (2, 122) = 53.631, *p* < 0.001, $\eta_{p}^{2}$ = 0.468. The results showed that US expectancy was significantly higher in BLOCK1 than in BLOCK2. In the post-hoc tests, the ratings for the DT were higher than the ratings for the CS+ (*MD* = 1.905, *SE* = 0.236, *p* < 0.001, 95% CI [1.433, 2.377]), and the ratings for the CS+ were significantly higher than the ratings for the CTX+ (*MD* = 0.636, *SE* = 0.245, *p* = 0.012, 95% CI [0.145, 1.126]). Additionally, the post-hoc tests revealed a significant difference between the ratings for the CS+ and the CTX+ in the stimulus bias group (*MD* = 1.058, *SE* = 0.352, *p* = 0.004, 95% CI [0.355, 1.761]). However, no significant difference was observed between the CS+ and the CTX+ in the context bias group (*MD* = 0.219, *SE* = 0.346, *p* = 0.530, the 95% CI [0.473, 0.911]). The interaction between block and image type was significant, *F* (4, 244) = 3.315, *p* = 0.011, $\eta_{p}^{2}$ = 0.052. The post-hoc tests indicated that the ratings for the CS+ were not significantly different from the ratings for the CTX+ in BLOCK1. Nevertheless, the ratings for the CS+ were significantly higher than those for the CTX+ in BLOCK2 and BLOCK3 (*MD* = 0.741, *SE* = 0.268, *p* = 0.007, 95% CI [0.206, 1.276]; *MD* = 0.575, *SE* = 0.242, *p* = 0.021, 95% CI [0.090, 1.060]) (see Figure 8A).

SCR: The results of a three-way repeated-measures ANOVA for image type (DT, CS+, CTX+) × group (stimulus bias, context bias) × block (BLOCK1, BLOCK2, BLOCK3) demonstrated a significant main effect of image type, *F* (2, 122) = 6.748, *p* = 0.002, $\eta_{p}^{2}$ = 0.100. The results of post-hoc tests indicated that the participants exhibited higher SCRs to the DT than that to the CTX+ (*MD* = 0.034, *SE* = 0.020, *p* = 0.001, 95% CI [0.014, 0.054]), and the SCRs to the CS+ were also significantly higher than those to the CTX+ (*MD* = 0.022, *SE* = 0.210, *p* = 0.030, 95% CI [0.002, 0.041]). In accordance with the results of US expectancy, only the stimulus bias group exhibited a significant difference between the CS+ and the CTX+ (*MD* = 0.036, *SE* = 0.014, *p* = 0.013, 95% CI [0.008, 0.063]), whereas no differences were observed between the ratings of the context bias group. The main effect of group was significant, *F* (1, 66) = 4.454, *p* = 0.039, $\eta_{p}^{2}$ = 0.068, with the participants in the stimulus bias group experiencing higher SCRs to threatening stimuli (see Figure 8B).

#### 3.4.4. Valence and Arousal Ratings

**Figure 9:**
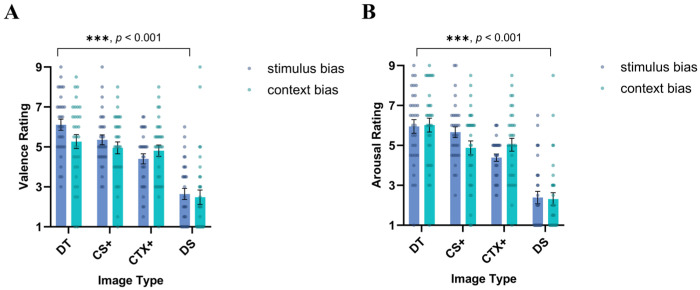
Valence and arousal ratings in Study 2. Results for both (**A**) valence ratings and (**B**) arousal ratings were significantly different for image type. The participants showed the highest ratings for the DT compared to the CS+ and CTX+ and the lowest ratings for the DS. Standard error bars are presented.

Valence ratings: A three-way repeated-measures ANOVA was conducted for image type (DT, CS+, CTX+, DS) × rating time (post-acquisition, post-extinction) × group (stimulus bias, context bias) on valence ratings. The results revealed a significant main effect of image type, *F* (3, 183) = 49.586, *p* < 0.001, $\eta_{p}^{2}$ = 0.448. Participants’ ratings for the DT were significantly higher than those for the CS+ (*MD* = 0.535, *SE* = 0.232, *p* = 0.024, 95% CI [0.072, 0.999]), and the ratings for the CS+ were significantly higher than those for the CTX+ (*MD* = 0.546, *SE* = 0.242, *p* = 0.027, 95% CI [0.063, 1.029]). Moreover, the ratings for the CTX+ were significantly higher than those for the DS (*MD* = 2.043, *SE* = 0.300, *p* < 0.001, 95% CI [1.442, 2.644]). In addition, only the stimulus bias group rated the CS+ higher than the CTX+ (*MD* = 0.952, *SE* = 0.344, *p* = 0.008, 95% CI [0.263, 1.640]), whereas the context bias group did not demonstrate a significant difference in ratings. A significant main effect of rating time was observed, *F* (1, 61) = 22.893, *p* < 0.001, $\eta_{p}^{2}$ = 0.273, with the participants providing significantly higher ratings post-acquisition than post-extinction. The interaction effect between image type and phase was significant, *F* (3, 183) = 2.739, *p* = 0.045, $\eta_{p}^{2}$ = 0.043. Post-hoc tests revealed that the DT, CS+, and CTX+ were rated significantly higher after the acquisition phase than post-extinction (*MD* = 1.073, *SE* = 0.388, *p* = 0.008, 95% CI [0.297, 1.849]; *MD* = 1.032, *SE* = 0.302, *p* = 0.001, 95% CI [0.428, 1.635]; *MD* = 1.499, *SE* = 0.271, *p* < 0.001, 95% CI [0.957, 2.040]), while DS ratings did not show a significant difference between the two phases (see Figure 9A).

Arousal ratings: A three-way repeated-measures ANOVA was conducted for image type (DT, CS+, CTX+, DS) × rating time (post-acquisition, post-extinction) × group (stimulus bias, context bias) on the arousal ratings. The main effect of image type was significant, *F* (3, 183) = 53.592, *p* < 0.001, $\eta_{p}^{2}$ = 0.468. The results indicated that the participants rated the DT significantly higher than the CS+ (*MD* = 0.715, *SE* = 0.269, *p* = 0.010, 95% CI [0.179, 1.252]), and that the CS+ was rated marginally higher than the CTX+ (*MD* = 0.559, *SE* = 0.283, *p* = 0.053, 95% CI [−0.008, 1.126]) and significantly higher than the DS (*MD* = 2.359, *SE* = 0.306, *p* < 0.001, 95% CI [1.748, 2.971]). Only the stimulus bias group rated the CS+ significantly higher than the CTX+ (*MD* = 1.274, *SE* = 0.404, *p* = 0.002, 95% CI [0.466, 2.082]) in the post-hoc tests, while no significant difference in ratings was exhibited in the contextual bias group. The main effect of rating time was also significant, *F* (1, 61) = 18.417, *p* < 0.001, $\eta_{p}^{2}$ = 0.232, indicating that the participants rated arousal significantly higher after the acquisition phase than post-extinction. The interaction effect between image type and rating time was significant, *F* (3, 183) = 3.617, *p* = 0.014, $\eta_{p}^{2}$ = 0.056. Post-hoc tests revealed that the ratings for the DS, CS+, and CTX+ were significantly higher post-acquisition than post-extinction (*MD* = 1.232, *SE* = 0.384, *p* = 0.002, 95% CI [0.465, 1.999]; *MD* = 0.685, *SE* = 0.315, *p* = 0.033, 95% CI [0.055, 1.316]; *MD* = 1.366, *SE* = 0.292, *p* < 0.001, 95% CI [0.781, 1.951]), which was the same as the valence ratings, whereas arousal ratings for the DS did not significantly differ between the two phases (see Figure 9B).

## 4. General Discussion

In order to explore the role of attentional bias on conditioned fear generalization, the present study employed an intervention designed to manipulate attentional bias in the experiment. Combining the methodologies of previous studies and the particularities of the present study, the participants in the present study were guided by attentional cue pictures to either global or local attention in the process of fear acquisition. The results of the study demonstrated the efficacy of the intervention. For those participants who focused on the global aspects in Study 1, there was no difference in either US expectancy or SCRs between the CS+ and CTX+ during fear acquisition. However, in Study 2, the stimulus bias group exhibited a stronger fear response to the CS+ as a consequence of directing the bias to the stimulus area, as reflected in the US expectancy results, as well as in the valence and arousal ratings. In contrast, the context bias group exhibited stronger fear towards the context stimulus in terms of US expectancy. The observed differences in acquisition between the different groups suggest that attentional bias interventions for participants are effective, and that different attention situations lead to different fear acquisition situations.

The impact of attentional bias on fear generalization was demonstrated by the differences observed between groups. In Study 1, there was no significant difference in the degree of generalization between the stimuli and context, indicating that the participants exhibited the same level of discrimination for the entire image. However, in Study 2, the stimulus bias group exhibited a lower degree of generalization towards stimuli and demonstrated a superior ability to discriminate the stimuli. Conversely, the context bias group had better discrimination of the context with lower fear generalization degree towards the context. Prior studies have also indicated that individuals’ degree of fear generalization is closely related to the breadth of attention. Furthermore, it has been demonstrated that focusing on a stimulus can effectively inhibit fear generalization [22,42]. This is also consistent with our findings showing that when individuals’ attention is biased towards a certain area, they will have a better ability to discriminate that area, which will produce a lower degree of fear generalization. It is evident that conditioned fear can generalize to both stimuli and context, and the extent of this generalization is also impacted by attention. Fear learning in the presence of attentional bias leads to heightened fear responses while simultaneously limiting the level of fear generalization in individuals.

A comparison of the results of Study 1 with those of Study 2 revealed that under conditions of attentional bias, individuals exhibited fear enhancement towards the stimulus. In Study 1, the participants who were attending to the global area did not exhibit differential fear responses to the stimulus and the context during fear extinction. In Study 2, the context bias group also demonstrated no significant differences in fear responses between the CS+ and CTX+ during the fear extinction phase, as did the ratings of valence and arousal for the CS+ and CTX+. However, in the stimulus bias group, the participants exhibited a stronger fear response to the CS+ during the fear extinction phase, both subjectively and physiologically. Furthermore, the participants who attention bias to the stimulus also felt stronger arousal to the CS+ and provided more negative ratings. This suggests that the condition of attention bias to the stimulus appeared to elicit markedly enhanced fear in response to the stimulus.

This may be attributed to the fact that the stimuli were positioned in the foreground position of vision, which is more likely to capture an individual’s attention and produce a foreground advantage [43]. Previous studies have demonstrated that the division of image regions into subjected and background has a profound effect on perception. Elements that are focal points of a subject’s attention undergo preferential processing and leave stronger memory traces [44,45], while the perceptual state of the background region is less clear. Other research indicates that background features may not be processed at the perceptual level [45]. It has also been proposed that background regions are actively suppressed [46,47,48]. When there is a foreground advantage and context inhibition, directing attention to a stimulus can significantly increase its perception and enhance the fear response. Another potential explanation is that the stimulus materials utilized in this experiment were photographs of human faces, a source with a great deal of information, with stronger relevance to the participants than the other stimuli [49]. Furthermore, individuals are more likely to exhibit attentional bias towards stimuli that are relevant to them [50]. Compared to all the landscape pictures utilized in the context section, faces represent highly relevant stimulus materials. Consequently, the stimuli will elicit a greater degree of attention, which will result in an individual exhibiting a more pronounced fear of the stimulus and an enhanced fear of the stimulus.

The results of the present study also indicated that the stimulus bias group was also found to have more difficulty extinguishing fear towards the CS+. In conjunction with Mogg’s finding, high anxiety is associated with faster attentional orienting and more challenging attentional extinction towards threatening stimuli [20]. Cognitive models of anxiety suggest that attentional bias towards threats may contribute to an anxious state [51]. The deep processing of stimuli by the stimulus bias group may have resulted in enhanced fear and difficulty extinguishing fear of the stimuli. The mechanisms involved in the formation of this fear can be further explored in future research.

In life, individuals exposed to fearful information exhibit a complex attentional situation. The findings of this study suggest that fear can generalize to both stimuli and context regardless of the presence of attentional bias. They also reveal that compared to global attention, attentional bias can distinguish the specific area of bias and cause a decreased level of fear generalization. Additionally, focusing on a stimulus heightens fear towards that particular area, a distinction not observed when attention is directed globally or towards the context. Variations in individuals’ attentional states result in varying levels of fear generalization and extinction, potentially contributing to the relatively low success rate of exposure therapy in clinical settings. Moving forward, clinical interventions targeting fear overgeneralization may benefit from tailoring treatment based on patients’ specific attentional biases when confronting fear, aiding in the alleviation of anxiety symptoms such as fear overgeneralization.

## 5. Limitation

Due to limitations in the experimental conditions, the participants recruited for this research were all healthy university students, with no clinical or subclinical participants included. Previous research has shown that individual anxiety levels and intolerance of uncertainty have significant relationships with fear generalization, yet the participants in this study did not show differences in those measures according to the questionnaire results. Future research could recruit participants in different states to further explore how varying levels of anxiety or intolerance of uncertainty influence the degree of fear generalization under different attention situations.

In addition, the homogeneity of the visual images is another limitation of this study. The context images only included scenic pictures and did not encompass other real-world scenarios. Furthermore, the facial images used as stimuli only featured male faces, which may introduce gender-related differences. Further research could consider using more diverse experimental materials and presentation methods to enhance the realism of both the contexts and stimuli.

## 6. Conclusions

The findings of the current study indicate that in situations involving conditioned fear, individuals generalize fear not only towards the stimulus but also towards the context. At the global level, there was no difference in fear responses between the stimuli and the context. However, participants’ discrimination ability improved as a result of the intervention of attentional bias instructions, which led to a reduction in the generalization towards that specific area. Furthermore, an attentional bias towards a stimulus is associated with a notable increase in fear towards the stimulus. These findings offer insights into the mechanisms underlying fear generalization, which may suggest new treatments for anxiety disorders. When addressing the overgeneralization of fear in individuals, it is important to consider an individual’s attention bias during fear acquisition in order to tailor interventions more effectively.

## Figures and Tables

**Table 1:** Overview of the characteristics of the participants in Study 1.

	*M ± SD*
Age (years)	19.973 ± 2.500
Shock tolerance (volts)	49.84 ± 10.658
STATE ANXIETY	29.135 ± 6.588
TRAIT ANXIETY	34.676 ± 4.854
IU	35.189 ± 5.211

The table includes information related to the age distribution, shock tolerance, state anxiety, trait anxiety, and intolerance of uncertainty (IU).

**Table 2:** Overview of the characteristics of the two participant groups in Study 2.

	Stimulus Bias Group	Context Bias Group	$\chi^{2}$	*t*	*p*
Gender (female/male)	19/12	21/10	0.157		0.500
Age (years)	20.56 ± 2.24	20.66 ± 2.09		0.731	0.468
Shock tolerance (volts)	53.48 ± 11.17	56.97 ± 11.84		1.201	0.235
STATE ANXIETY	43.94 ± 11.07	39.38 ± 10.50		1.678	0.269
TRAIT ANXIETY	46.03 ± 9.76	40.41 ± 9.91		2.270	0.080
IU	28.74 ± 6.87	29.78 ± 6.60		0.612	0.543

The table includes information related to the gender ratio, age distribution, shock tolerance, state anxiety, trait anxiety, and intolerance of uncertainty. No significant differences were observed between the two groups across these characteristics, indicating their comparability for the study.

## Data Availability

The data of this study are available from the corresponding author upon reasonable request.
